# Molecular dynamics simulations of human α-defensin 5 (HD5) crossing gram-negative bacterial membrane

**DOI:** 10.1371/journal.pone.0294041

**Published:** 2023-11-21

**Authors:** Tadsanee Awang, Phoom Chairatana, Prapasiri Pongprayoon

**Affiliations:** 1 Department of Chemistry, Faculty of Science, Kasetsart University, Bangkok, Thailand; 2 Department of Microbiology, Faculty of Medicine Siriraj Hospital, Mahidol University, Bangkok, Thailand; 3 Center for Advanced Studies in Nanotechnology for Chemical, Food and Agricultural Industries, KU Institute for Advanced Studies, Kasetsart University, Bangkok, Thailand; Nanyang Technological University, SINGAPORE

## Abstract

Human α-defensin 5 (HD5) is a cationic antimicrobial peptide exhibiting a wide range of antimicrobial activities. It plays an important role in mucosal immunity of the small intestine. HD5 exerts its bactericidal activities through multiple mechanisms, one of which involves HD5 inducing the formation of pores in the bacterial membrane, subsequently allowing the peptide to enter the bacterial cytoplasm. Nevertheless, the precise molecular intricacies underlying its bactericidal mechanisms remain inadequately understood. In this work, the Potential of Mean Force (PMF) was computed to delve into the energetic properties governing the movement of HD5 across the lipopolysaccharide (LPS) membrane, which is a representative model of the gram-negative bacterial membrane. Our findings indicate that the most favorable free energy is attained when HD5 binds to the surface of the LPS membrane. This favorable interaction is primarily driven by the strong interactions between arginine residues in HD5 and the charged head groups of LPS, serving as the predominant forces facilitating the adhesion of HD5 to the membrane. Our analysis reveals that a dimeric form of HD5 alone is sufficient to create a water-filled channel in the membrane; however, achieving the complete lysis of the gram-negative bacterial membrane requires higher-order oligomerization of HD5. Our results suggest that HD5 employs the toroidal pore formation mechanism to disrupt the integrity of the LPS membrane. Furthermore, we identified that the primary energy barrier obstructing HD5 from traversing the membrane is localized within the hydrophobic core of the membrane, which is also observed for other defensins. Additionally, our study demonstrates that a mixture of HD5-LPS leads to a thinning of the membrane. Taken together, this work provides a deeper insight into the molecular intricacies governing the behavior of HD5 as it translocates through the gram-negative bacterial membrane.

## Introduction

Antimicrobial peptides (AMPs) are small naturally occurring peptides that play a crucial role in the innate immune system of various organisms, including humans. AMPs exhibit a wide array of inhibitory effects against invading pathogens [1]. Some AMPs possess the ability to directly eliminate pathogens, whereas others exert immunomodulatory functions [2]. These peptides are typically comprised of 10–60 amino acids and are positively charged. These distinctive characteristics make AMPs promising candidates for the development of novel antibiotics. Several naturally occurring AMPs, such as nisin, gramicidin, and polymyxins, have already found their way into the market. Moreover, some AMPs are currently undergoing clinical trials, such as calthelicidin LL-37 [3].

Defensins are small host-defense peptides with a molecular weight ranging from 3.5 to 6 kDa. They are further classified into three groups: α-, β-, and θ-defensins, based on the distribution of cysteine residues and disulfide-bond linkages [4]. Nevertheless, in humans, only α- and β-defensins are present [5]. Currently, six human α-defensins have been identified: human neutrophil peptide 1–4 (HNP1-4), and human defensin 5 and 6 (HD5, and HD6) [4]. HNP1-4 are predominantly found in neutrophils, whereas HD5 and HD6 are produced by Paneth cells in the intestinal crypts, where they serve as protective agents for the small intestine against microbial pathogens. Among these defensins, HD5 has garnered significant attention and research. The peptide demonstrates remarkable efficacy against a broad spectrum of gram-negative bacteria through various mechanisms of action. For instance, HD5 can disrupt and cause damages to the gram-negative bacterial membrane by inducing pore formation, and subsequently penetrating into the bacterial cytoplasm [1]. Furthermore, HD5 exhibits anti-endotoxin properties by binding to bacterial lipopolysaccharide (LPS), thereby neutralizing its effects [6, 7]. Numerous experimental studies have been conducted to elucidate the impact of HD5-LPS interactions on microbial cell activity [1, 8, 9], yet microscopic details remain elusive.

**Fig 1:**
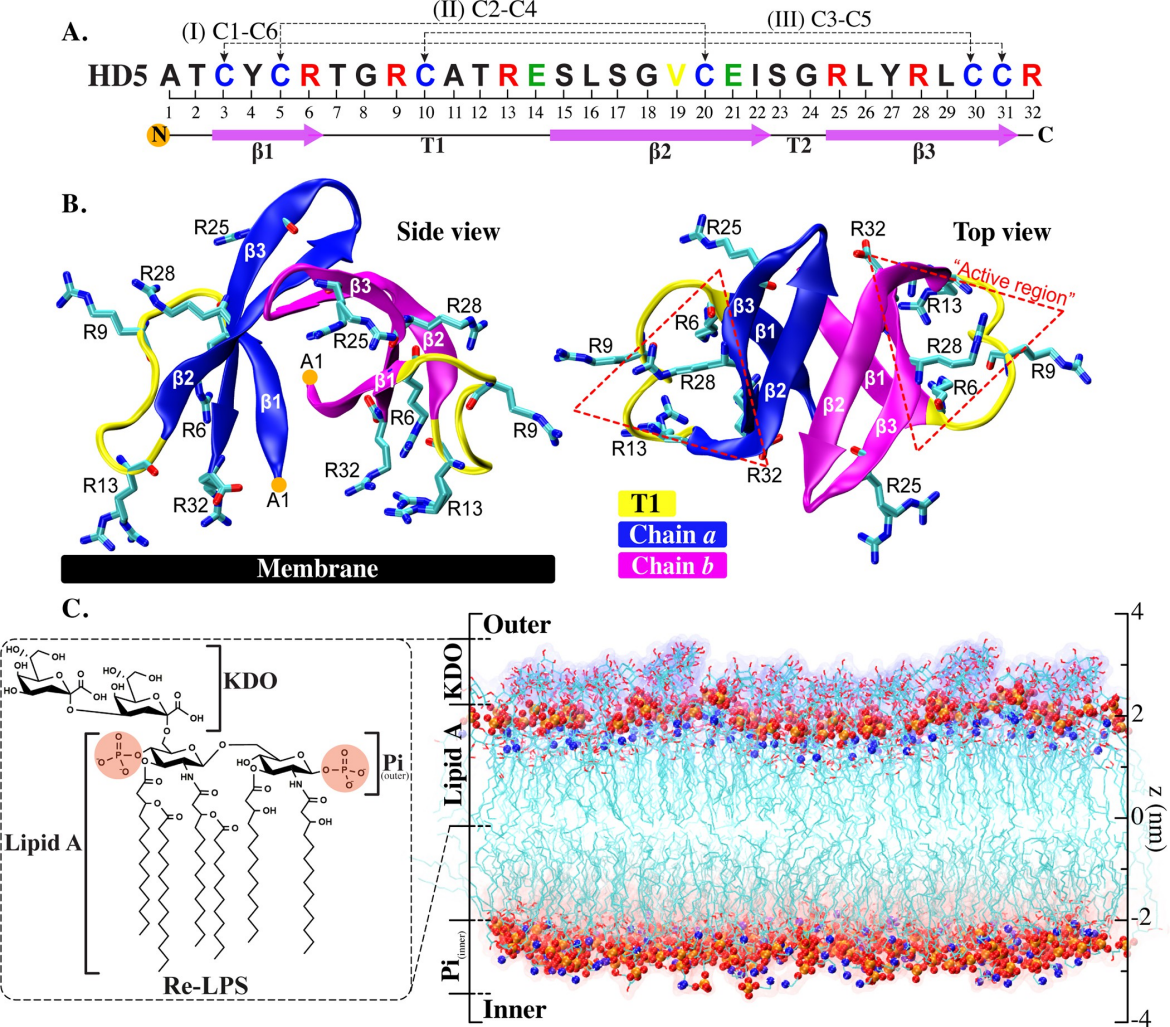
HD5 structure and gram-negative bacterial membrane. A. An amino acid sequence of HD5 where disulfide bonds and secondary structures are labeled. Conversed cysteine and valine residues are in blue and yellow, whereas positively charged arginine and negatively charged glutamate residues are in red and green, respectively. B. Side and top views of an HD5 dimer. The arginine-rich active region is shown in dotted red triangles. The flexible loop T1 is displayed in yellow. C. A simplified model of the lipopolysaccharide (LPS) membrane (Re-LPS) used in this work. Only 3-deoxy-D-*manno*-octulosonic acid (KDO) and lipid A are involved in this outer membrane.

HD5 consists of 32 amino acids with a total charge of +4 at neutral pH and is rich in arginine (R) residues and hydrophobic residues [10]. Its cationic nature, along with a distinct arrangement of charged residues on its surface, may contribute to its exceptional bactericidal potency among the six human α-defensins [11]. The structure of HD5 comprises a three-stranded β-sheet core stabilized by three intramolecular disulfide bonds (C1-C6, C2-C4, and C3-C5). HD5 exhibits an amphipathic character due to the presence of both hydrophilic and hydrophobic residues. A dimeric configuration of HD5 has been documented as the most functionally active form, wherein each monomer is linked through a β2 strand (Fig 1A and 1B), while an HD5 tetramer is considered to be the largest oligomeric state [11, 12]. A dimeric HD5 contains the arginine-rich active region (R25, K26, Y27, and R28), which is located near the C-terminus (Fig 1B). Both the amphipathic nature of HD5 and the presence of this active region are imperative for the potent antibacterial activity exhibited by HD5 [12, 13]. Consequently, HD5 stands as one of the most well-characterized defensins, and hence, this host-defense peptide is a promising candidate for the development of novel antibiotics.

In the scope of this study, the energetic properties governing the permeation of HD5 through the gram-negative bacterial membrane was investigated by using the umbrella sampling (US) technique. The Potential of Mean Force (PMF) was measured along the permeation pathway. While previous studies have provided insights into how HD5 associates with the membrane surface and resides within the membrane at different positions [14–16], they have not unveiled the mechanisms underlying protein penetration or pore formation. Thus, our study endeavors to unravel the intricate mechanisms through which HD5 induces pore formation on the bacterial membrane, adapts its conformation, and travels through the bacterial membrane. A simplified bacterial LPS membrane was used to represent the gram-negative bacterial membrane in this work (Fig 1C). The insights into HD5 permeation mechanisms gained from this study are anticipated to be instrumental in the future design of HD5-mimicking antimicrobial peptides aimed at combatting bacterial infections.

## Materials and methods

### Potential of Mean Force (PMF) of HD5 insertion

The initial configuration of HD5 bound to the LPS membrane was extracted from our previous work [14]. The topological parameters of a simplified LPS model (Re-LPS), which encompasses lipid A and 3-deoxy-D-*manno*-octulosonic acid (KDO), were adopted from a prior investigation [17]. For the outer membrane (OM) model used here, the outer leaflet comprises simplified lipopolysaccharide (LPS) model (Re-LPS), which is neutralized by magnesium ions (Mg^2+^). In contrast, the inner leaflet consists of 90% 1-palmitoyl 2-cis-vaccenic phosphatidylethanolamine (PVPE), 5% 1-palmitoyl 2-cis-vaccenic phosphatidylglycerol (PVPG), and 5% 1-palmitoyl 2-cis-vaccenic 3-palmitoyl 4-cis-vaccenic diphosphatidyl-glycerol (PVPV DPG) [17, 18].

The reaction pathway was defined along the membrane axis (z direction) and the reaction coordinate was modulated by adjusting the extent of insertion of HD5 into the LPS membrane, with an increment of 0.1 nm. In the initial window, an HD5 dimer was positioned in the bulk solution above the surface of the LPS membrane. The initial structures for each umbrella window were generated by subjecting HD5 to a pulling force along the reaction pathway with a spring constant of pulling force (k) of 1,000 kJ mol^-1^ nm^2^ and a pulling velocity (*v*) of 0.8 nm ns^-1^. These pulling parameters were adapted from a previous study on membrane-penetrating AMP [19]. For each window, the HD5 dimer was displaced by 0.1 nm into the bilayer and restrained in that position. The range of interest spanned form approximately -5.4 nm to +5.1 nm, divided into 92 windows, and the total simulation time amounted to roughly 9 μs.

All molecular dynamics (MD) simulations were executed employing GROMACS5.0 package [20] with GROMOS 53A6 force fields [20]. HD5, the membrane, and the salt solution were coupled separately. At each restrained position (window), the system underwent energy minimization through 10,000 steps to remove bad contacts. Subsequently, each window was equilibrated for 10 ns and followed by a 100-ns production run at 323 K and 1 atm. The Potential of Mean Force (PMF) was computed based on data collected over the 100-ns production runs and was constructed using the weighted histogram analysis method (WHAM) in the GROMACS package [21]. The temporal overlap was assessed via probability distribution (S1 and S2 Figs in Supplementary Information). The Particle Mesh Ewald (PME) method [22] was applied for electrostatic treatment with a short-range cutoff of 1 nm and a Fourier spacing of 0.12 nm. A 2-fs integration time step was employed with LINCS algorithms [23]. Simulations were conducted in the NPT ensemble (constant number of particles, pressure, and temperature) employing a semi-isotropic Parrinello-Rahman barostat with τ_p_ = 1 ps and a v-rescale thermostat [16] with a coupling constant of τ_p_ = 0.1 ps.

All results were analyzed by GROMACS tools and in-house codes. Hydrogen bonds were computed using g_hbond with default parameters, setting the hydrogen–donor–acceptor cutoff angle to 30° and the cutoff radius (X-acceptor) to 0.35 nm. The root-mean-square deviations (RMSDs) and fluctuations (RMSFs) were computed in relation to an initial structure at 0 ns from each production run, serving as a reference. Visualizations and molecular graphic images were prepared using Virtual Molecular Dynamics software (VMD 1.9.4) [24].

## Results and discussion

**Fig 2:**
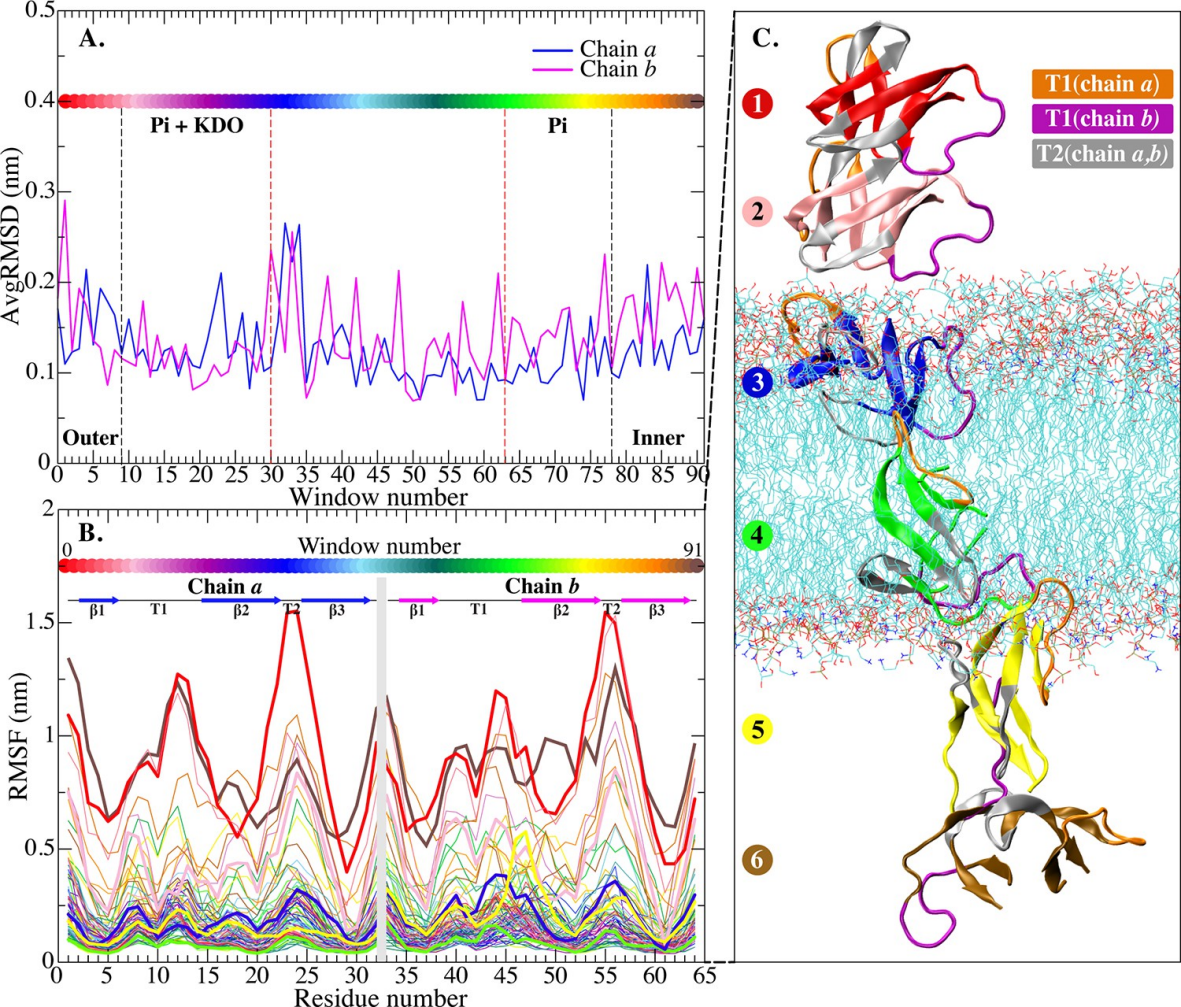
Stability and fluctuation of HD5 at all positions. A. C-alpha RMSDs of each HD5 chain from each window. B. C-alpha RMSFs of chain *a* and chain *b* of HD5. The secondary structures of each residue are also shown in B. The color gradient bands in A and B indicate the HD5 position across the membrane (window numbers from 0 (extracellular side) to 91 (intracellular side)). All RMSDs and RMSFs are computed using an initial structure at t = 0 ns as a reference. C. Different positions of an HD5 dimer across the membrane are shown in different colors based on the window numbers: T1 (chain *a*), T1 (chain *b*), and T2 (chain *a*, *b*) loops are highlighted in orange, magenta, and gray, respectively.

In order to gain insights into the structural and dynamic characteristics of HD5 during its permeation through the membrane, we initially assessed the structural properties of an HD5 dimer by analyzing root-mean-square deviations (RMSDs) and fluctuations (RMSFs) (Fig 2). In Fig 2A, it is apparent that chain *b* exhibits a higher degree of fluctuation compared to chain *a*. Nevertheless, both chains retain a high level of flexibility as the dimer approaches the interface between the LPS layer and the hydrophobic region of membrane (Fig 2A). Overall, HD5 displays high RMSFs when in the bulk solution, particularly at the T1- and T2-loop regions, as indicated by the red and brown lines in Fig 2B. We speculate that the high RMSF values are attributed to the unrestricted mobility of the peptide in the solution.

In contrast, the HD5 insertion into the LPS membrane induces a notable enhancement in the structural rigidity of the HD5 structure (Fig 2B). While the HD5 dimer retains some degree of flexibility during adsorption onto the LPS layer, particularly within the T1 and T2 regions, the dimer becomes progressively more rigid upon reaching the hydrophobic core (Fig 2B and 2C). The high mobility of T1 and T2 loops aligns with previous studies highlighting the significance of these regions in facilitating HD5 to contact with LPS [14, 15, 25]. The orientations of HD5 along the reaction coordinates are shown in Fig 2C, with the T1 and T2 loops of each chain being explicitly labeled. A more detailed examination of the dynamics exhibited by the T1 and T2 loops is shown in S3 Fig in Supplementary Information.

**Fig 3:**
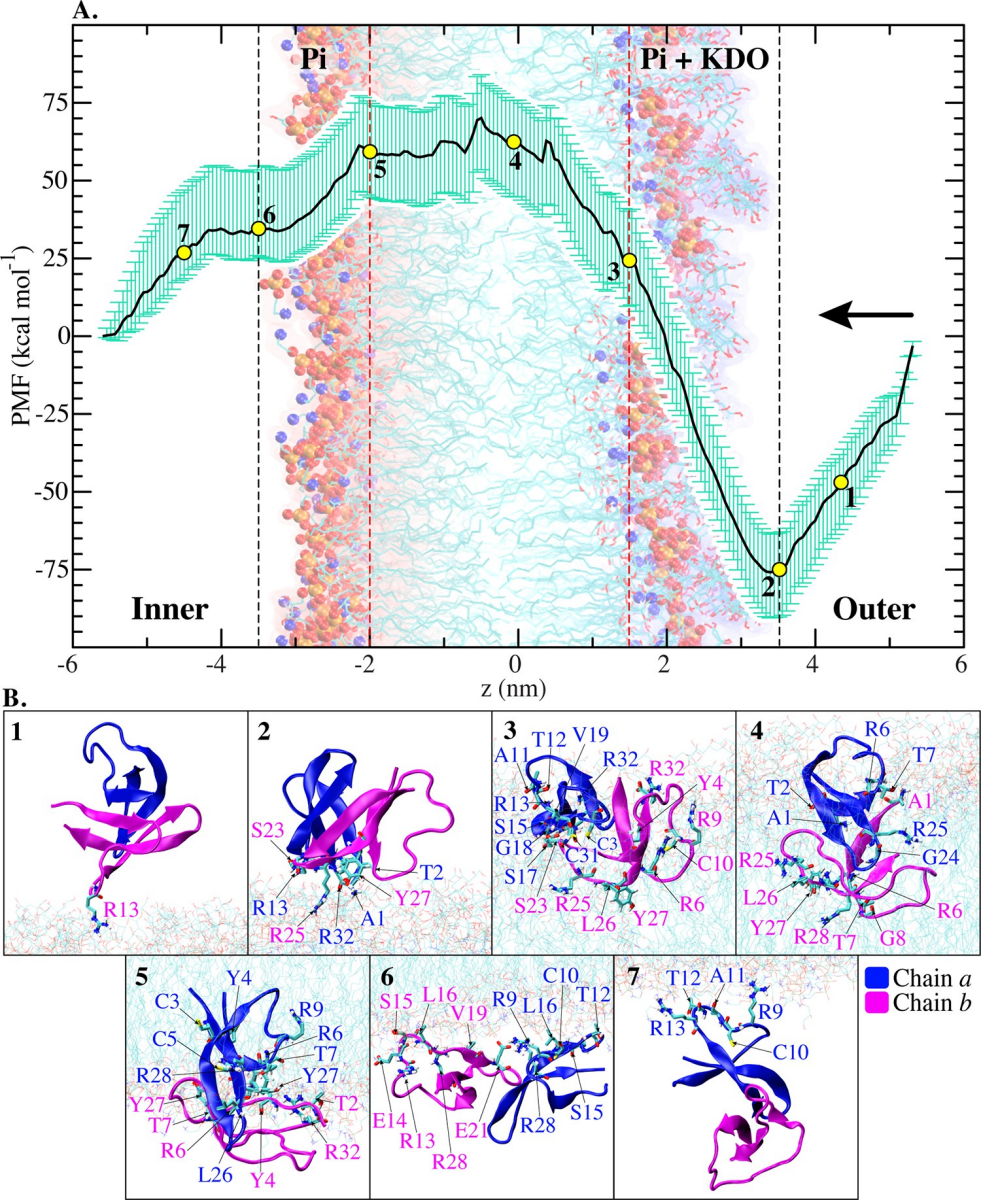
Potential of Mean Force and HD5 orientations. A. The Potential of Mean Force (PMF) is shown with bootstrap error bars (green) for HD5 insertion into the LPS membrane. The starting position of HD5 is on the right side and it moves along the x-axis to the left side (indicated by an arrow for the direction). Numbers “1–7” correspond to key positions in the PMF, where the HD5 orientations and key residues involving in LPS-HD5 interactions are depicted in B. Chains *a* and *b* are highlighted in blue and magenta, respectively.

To assess the free energy associated with the insertion of HD5 into the LPS membrane, the Potential of Mean Force (PMF) was computed (Fig 3A). Notably, the surface-bound state of HD5 (position 2) exhibits lower energy compared to HD5 residing in the bulk solution (position 1). This observation underscores a robust driving force compelling HD5 to adsorb onto the LPS membrane. As anticipated, a distinct free energy well is evident at the LPS surface (position 2 in Fig 3A), which actively propels the HD5 dimer to approach and remain close to the charged LPS surface. This favorable free energy pattern agrees with findings in other antimicrobial peptides [26–28]. The pronounced favorability of this free energy confirms that the LPS layer represents the primary barrier for HD5 to penetrate into the cytoplasm, which is consistent with prior studies [15, 25].

The energy barrier escalates as HD5 embarks on its journey into the membrane core (positions 2 to 5). The maximum unfavorable energy is reached when HD5 reaches the hydrophobic core (position 5). Unlike β-defensins, where their dimers dissociate during membrane adsorption, the HD5 dimeric state remains intact upon adsorption onto the LPS membrane [29]. Furthermore, analogous to other cationic antimicrobial peptides [27, 30, 31], the hydrophobic core emerges as an unfavorable region for HD5, which is supported by our energy barrier data spanning positions 3 to 5 in Fig 3A. This energy barrier, approximately 60 kcal/mol in magnitude, is comparable to that observed in the permeation of *β*-defensin 3 [32].

We also observe a stretching phenomenon in HD5 while it resides in the hydrophobic region of the membrane. Nevertheless, its structure stabilizes once it departs from this hydrophobic zone (S3 Fig in Supplementary Information). When HD5 exits the hydrophobic core, repulsive interactions are reduced, particularly as the peptide is pushed toward the inner charged (Pi) surface (positions 5–6 in Fig 3A and 3B).

In summary, the penetration of HD5 into the asymmetrical LPS membrane unfolds into three primary stages: (I) A cationic HD5 dimer swiftly adsorbs onto the LPS membrane through interactions with negatively charged LPS moieties; (II) The HD5 dimer encounters a formidable energy barrier, which hinders its translocation across the hydrophobic core; (III) Upon surpassing this energy barrier, HD5 proceeds further toward the intracellular side, following a downhill energetic pathway. Further in-depth analysis will be discussed in subsequent sections.

**Fig 4:**
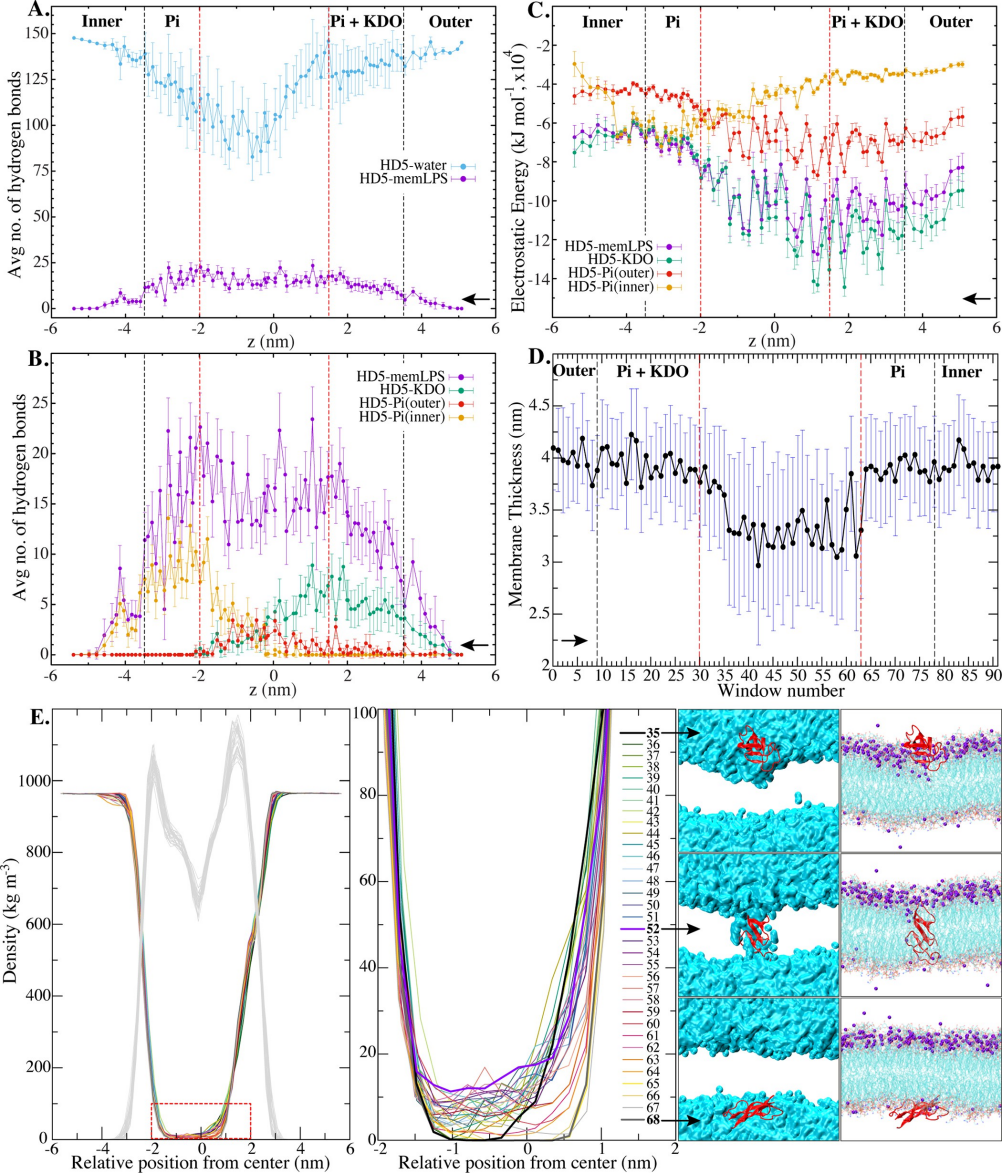
Interactions and membrane properties. A. Average number of hydrogen bonds involving HD5 with water molecules and the whole membrane (HD5-water and HD5-memLPS). B. Average number of hydrogen bonds between HD5 and key components, namely memLPS, KDO, Pi(outer), and Pi(inner). C. Electrostatic energies between HD5 and key components, namely memLPS, KDO, Pi(outer), and Pi(inner). The black arrow indicates the direction of HD5 translocation across the membrane. All data are presented with standard deviation. D. Membrane thickness as a function of umbrella windows with standard deviation. The thickness is computed based on the distances between Pi from both leaflets. E. Density distribution of water molecules as a function of umbrella window and the LPS membrane (shown in gray), with the degrees of water leakage in the membrane magnified in an inset. Water leakage and key positions of HD5 inside the membrane are displayed on the right. The cyan surface represents water, and HD5 is shown in red. Mg^2+^ ions are labeled in purple.

In addition to assessing the energetic aspects, we also conducted a comprehensive investigation into the interactions between HD5 and the LPS membrane. As demonstrated in Fig 4A, a dimeric HD5 consistently forms hydrogen bonds with the polar constituents of the membrane throughout its translocation pathway. Furthermore, HD5 appears to entrain water molecules, potentially leading to the formation of a water-filled channel, as illustrated in Fig 4E. Nonetheless, it is worth noting that the lowest number of water contacts is observed when HD5 reaches the center of the hydrophobic core (Fig 4A).

The quantitative evaluation of hydrogen bonds, as presented in Fig 4B, emphasizes the significant role of KDO at the LPS surface in facilitating the insertion of HD5 into the membrane. During the translocation of HD5, the peptide effectively carries KDO into the membrane core, where it maintains some interactions with Pi at the outer leaflet. The presence of KDO seems to potentiate the electrostatic environment within the hydrophobic core, which promotes the influx of water molecules into this region. Our data underscore electrostatic interactions as the predominant driving force governing the translocation of HD5 across the gram-negative bacterial membrane (Fig 4C). Interactions between HD5 and the Pi moieties at the inner membrane are also crucial for facilitating its translocation to the inner leaflet zone. As HD5 reaches the midpoint of the membrane, it can engage with charged moieties on both sides, including KDO, Pi (outer), and Pi (inner). This observation highlights the indispensable nature of an electrostatic environment for the insertion of HD5 into the membrane. The comparable magnitudes of electrostatic energies between HD5-memLPS (the entire membrane) and HD5-KDO support the essential role of the KDO moieties in the attachment and subsequent insertion of HD5 (Fig 4C). The potent electrostatic energies between HD5 and KDO also elucidate the necessity for HD5 to be accompanied by KDO during the insertion process. Moreover, HD5 must surmount the deep energy well associated with KDO to complete the membrane penetration process.

Our investigation also reveals that the penetration of HD5 not only disrupts the LPS surface, but also breaches the membrane, leading to membrane thinning (Fig 4D), along with membrane curvature (S3 Fig in Supplementary Information). Nonetheless, after HD5 penetrates the cytoplasm, membrane recovery begins, and deformation of the water-filled channel is observed (Fig 4A and 4E). The presence of a water-filled cavity within the LPS membrane is shown in Fig 4E. Throughout the translocation of HD5 across the LPS membrane, the maximum water-holding capacity is achieved when HD5 is positioned at the midpoint of the membrane (Fig 4E). This observation suggests that the accumulation of HD5 inside the membrane is required to effectively disrupt membrane stability. When an HD5 dimer resides within the hydrophobic core in proximity to the Pi(inner) region (Z ~ -0.5 nm) (Fig 4B), HD5-water interactions are maximized. These interactions with water serve to stabilize HD5 when it is situated within the membrane core (Fig 4A and 4E). Nevertheless, it is important to note that the formation of a water-filled channel during the translocation of HD5 appears to be transient, suggesting that the cytolytic activity of HD5 requires a high concentration of the peptide, even though HD5 penetration is more favorable at lower concentrations.

**Fig 5:**
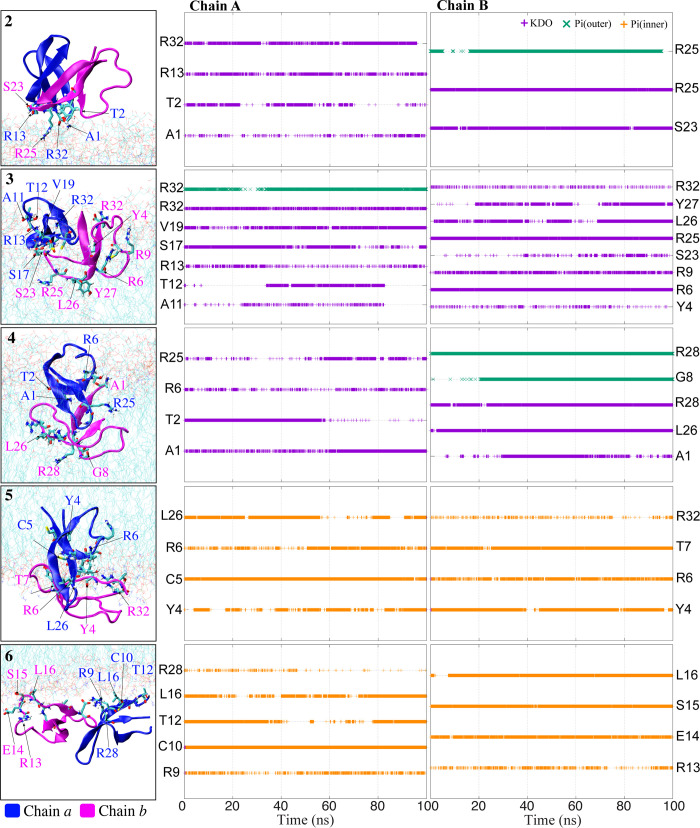
Number of hydrogen bonds between HD5 and LPS membrane at positions 2–6 as a function of time. The HD5-membrane locations can be seen on the left. Purple, green, and orange plots stand for KDO, Pi(outer), and Pi(inner), respectively. The key residues involving in the interactions between HD5 and the LPS membrane are labeled in the y axis. Chains *a* and *b* are displayed in blue and magenta, respectively.

Furthermore, we conducted a comprehensive analysis of the secondary structural changes in HD5 at various positions (1–7) during its membrane permeation process, which was then compared to its X-ray structure (Table 1). The structural elements presented in Table 1 focus exclusively on β-sheet and turns, while comprehensive plots detailing the secondary structure of HD5 at these positions are provided in S4 Fig in Supplementary Information. Our investigation reveals that the primary β-sheet structures of HD5 remain stable throughout the course of its membrane translocation. Nonetheless, a minor stretching of HD5 is observed when the peptide resides within the hydrophobic core (position 4), approximately 63.89% (Table 1). This slight elongation of HD5 is speculated to be associated with its interaction with the KDO and Pi(outer) components of the LPS surface, as depicted in Fig 5 and S3 Fig in Supplementary Information. Importantly, it should be noted that the secondary structure of HD5 returns to its original state once it reaches the inner surface (position 5), approximately 75% (Table 1).

**Table 1:** Secondary structures of HD5 at different positions across a LPS membrane. The percentage of coils is not shown here.

Position	Secondary Structure		
	Structure	β-sheet	Turn
**X-ray**	100.00	83.33	16.67
**1**	79.17	70.83	6.95
**2**	84.72	76.39	8.34
**3**	73.61	63.89	9.72
**4**	63.89	51.39	8.34
**5**	75.00	65.28	8.34
**6**	81.94	70.83	11.11
**7**	80.56	72.22	6.95

To further elucidate the penetration mechanism of HD5, we performed an analysis of hydrogen bond formation between the LPS membrane and key residues at positions 2–6 are computed (Fig 5). Only residues involved in strong interactions are considered for this analysis. At position 2, when HD5 reaches the LPS surface, it adopts an orientation nearly perpendicular to the membrane surface (Fig 5). This arrangement allows certain residues, including A1, T2, R13, and R32 in one chain, and S23, R25 in the other chain, to interact with the KDO moieties. Furthermore, R25 in chain *b* is able to penetrate deeper into the LPS core and interact with the Pi(outer) components (Fig 5). At position 3, HD5 lies on the LPS surface and gets encased by polar moieties. This positioning allows polar residues, namely A11, T12, R13, S17, V19, and R32 in chain *a*, and Y4, R6, R9, S23, R25, L26, Y27, and R32 in chain *b*, to form multiple hydrogen bonds with the membrane (Fig 5). Arginine residues appear to be the major contributors to the interactions between HD5 and the membrane. Our finding is in accordance with a previous study, which demonstrated that replacing non-cationic amino acids with cationic arginine residues significantly enhances the antibacterial activity of HD5 [33]. In addition, we observe that KDO plays a substantial role in the adsorption of HD5, consistent with prior studies [14, 15, 25]. Notably, no dissociation of the HD5 dimer is observed during its adsorption process.

When HD5 reaches the hydrophobic core (position 4), its dimeric state becomes destabilized due to the loss of hydrogen bonds with the LPS components (Fig 5), resulting in the unfavorable free energy observed in Fig 3A. This finding aligns with a previous experimental study that demonstrated the antagonistic interaction of HD5 with LPS [8]. Moreover, we observe that the penetration of HD5 into the membrane leads to water leakage, as previously reported [15]. Nevertheless, the loss of hydrogen bonds between HD5 and the LPS moieties is partially compensated by the interactions with water molecules accompanying HD5 (Fig 4A and 4E). Nevertheless, these interactions are insufficient to maintain HD5 inside the hydrophobic core. Concerning the orientation of HD5 at position 4, chain *a* remains on the surface, making contact with the KDO moieties through its A1, T2, R6, and L25 residues, while chain *b* shifts downward toward the center of the membrane and interacts with the Pi(outer) moieties using its A1, G8, L26, and R28 residues (Fig 5, left). Additionally, some residues can interact with the fatty acid portion of lipid A in LPS (S5 Fig in Supplementary Information). As HD5 reaches the Pi(inner) side (position 5), both chains interact with the Pi groups on the inner leaflet through residues Y4, C5, R6, T7, L26, and R32. At this position, a significant portion of chain *a* remains embedded within the hydrophobic core (Fig 5). At position 6, where both chains are exposed to the bulk solution, they anchor to the inner leaflet by interacting with the Pi(inner) moieties via residues R9, C10, T12, R13, E14, S15, L16, and R28 (Fig 5). These findings indicate that most of the residues required for the adhesion of HD5 to the Pi(inner) surface are in the T1 region (residues 6–14 in Fig 1A). Furthermore, this T1 region also plays an essential role in interacting with the membrane when the peptide is situated in polar regions, such as the LPS surface and Pi(inner). Nevertheless, the significance of this T1 loop diminishes once HD5 enters the hydrophobic core. Based on our data, we postulate that the T1 loop primarily contributes to the formation of interactions with charged residues on the membrane surface. This observation also explains the high flexibility of the loop when HD5 is present in both outer and inner leaflets (Fig 2B).

In comparison to our previous molecular dynamics (MD) work on HD5 [14, 15, 25], although we did not identify specific residue responsible for membrane binding, our current study still underscores the crucial role of the T1 loop in attaching HD5 to the membrane surface. Moreover, we observe similar orientations of HD5 during its traversal across the membrane. The HD5 dimer initiates its membrane permeation process by placing one of its monomers on the membrane surface and penetrating the LPS membrane by using that monomer. During the penetration, HD5 also pulls along the polar head groups of the LPS layer, resulting in the formation of a water-filled channel. This event also disrupts the membrane stability, demonstrating that an HD5 dimer is capable of creating a water channel within the LPS membrane. This “dimer pore” topology is consistent with observations in other defensins [29], such as human α-defensin 1 [34]. Nonetheless, the water-filled channel dissipates once the HD5 dimer is expelled from the membrane.

## Conclusion

In this study, we have conducted a comprehensive investigation into the structural and energetic aspects of HD5 permeation through the gram-negative bacterial membrane. The primary driving forces behind the interaction between HD5 and the membrane are the electrostatic interactions between its arginine residues and the negatively charged components of the membrane. These electrostatic interactions not only contribute to the target specificity of this peptide, but also serve as the means by which HD5 anchors itself to the LPS membrane. Furthermore, the presence of an HD5 dimer within the membrane leads to membrane thinning, and ultimately the formation of a water-filled channel. This observation underscores the notion that the dimeric conformation of HD5 is adequate to create a water channel in the membrane. During its penetration into the membrane, HD5 interacts with and draws the polar head groups of lipid A, a constituent of LPS, into the membrane core. This process results in alterations in membrane thickness and initiates the water leakage. Consequently, lipid A pulling the polar head groups into a membrane core emerges as one of the key mechanisms by which HD5 disrupts the structural integrity of gram-negative bacterial membrane. This insertion of LPS head groups suggests the toroidal-pore model as a plausible mode of action for HD5. Nonetheless, the extrusion of HD5 to the surface of the inner leaflet can deform the water-filled channel, allowing the membrane to recover from the damage caused by HD5. This observation implies that the lysis of gram-negative bacterial membrane necessitates the accumulation of HD5 molecules within the membrane core, akin to what has been reported for other cationic antimicrobial peptides [35, 36]. In addition, our findings reveal that strong electrostatic interactions between HD5 and KDO present a significant barrier to the penetration of HD5 into the cytoplasm. In summary, this work provides valuable mechanistic insights into how HD5 disrupts gram-negative bacterial membrane. These insights hold promise for the development of HD5-derived antibacterial peptides tailored for the treatment of gram-negative bacterial infections.

## Supporting information

S1 Fig Probability distribution. The range is from ~ -5.4 nm to +5.1 nm, which is divided into 92 windows.
(TIF)

S2 Fig PMF profiles. The plots are generated from the data in ranges of 0–40 ns, 20–60 ns, 40–80 ns, and 60–100 ns, respectively.
(TIF)

S3 Fig Orientations and locations of HD5 at different stages of its membrane penetration. Top: Superimposition of six final snapshots with the X-ray structure of HD5 (PDB ID: 1ZMP, transparent cyan). Bottom: Six stages of HD5 translocation through the LPS membrane. The peptide is shown in cartoon representation, and the lipid head groups are shown in a VDW representation. The lipid tails are shown as lines. The semi-transparent blue and red surfaces correspond to the KDO moieties, Pi(outer) and Pi (inner), respectively. The arrow indicates the direction in which the pulling force is applied: (1) The initial system. (2) The peptide binds to the surface of the outer membrane layer. (3) The peptide buries through the barrier layer of KDO. (4) The peptide reaches the center of the membrane bilayer. (5) The peptide leaves the surface of the inner membrane layer. (6) The peptide is out of the membrane.
(TIF)

S4 Fig Secondary structures of HD5 at 7 positions. Positions 1–7 are the same as shown in Fig 3B.
(TIF)

S5 Fig Number of hydrogen bonds between fatty acid moieties of Lipid A and HD5. A chemical structure of lipid A is shown as an inset where fatty acid moieties are shown in green band.
(TIF)

## References

[1] Chileveru HR, Lim SA, Chairatana P, Wommack AJ, Chiang IL, Nolan EM. Visualizing attack of Escherichia coli by the antimicrobial peptide human defensin 5. Biochemistry. 2015;54(9):1767–77. doi: 10.1021/bi501483q 25664683 PMC5270551

[2] Huan Y, Kong Q, Mou H, Yi H. Antimicrobial Peptides: Classification, Design, Application and Research Progress in Multiple Fields. Front Microbiol. 2020;11:582779. doi: 10.3389/fmicb.2020.582779 33178164 PMC7596191

[3] Reinholz M, Ruzicka T, Schauber J. Cathelicidin LL-37: an antimicrobial peptide with a role in inflammatory skin disease. Ann Dermatol. 2012;24(2):126–35. doi: 10.5021/ad.2012.24.2.126 22577261 PMC3346901

[4] Chen HQ, Xu ZN, Peng L, Fang XM, Yin XF, Xu NZ, et al. Recent advances in the research and development of human defensins. Peptides. 2006;27(4):931–40. doi: 10.1016/j.peptides.2005.08.018 16226346

[5] Pazgier M, Li X, Lu W, Lubkowski J. Human defensins: synthesis and structural properties. Curr Pharm Des. 2007;13(30):3096–118. doi: 10.2174/138161207782110381 17979752

[6] Zhang Y, Cougnon FB, Wanniarachchi YA, Hayden JA, Nolan EM. Reduction of human defensin 5 affords a high-affinity zinc-chelating peptide. ACS Chem Biol. 2013;8(9):1907–11. doi: 10.1021/cb400340k 23841778 PMC3783636

[7] de Leeuw E, Burks SR, Li X, Kao JPY, Lu W. Structure-dependent functional properties of human defensin 5. FEBS Lett. 2007;581(3):515–20. doi: 10.1016/j.febslet.2006.12.036 17250830 PMC1832120

[8] Wang C, Shen M, Zhang N, Wang S, Xu Y, Chen S, et al. Reduction Impairs the Antibacterial Activity but Benefits the LPS Neutralization Ability of Human Enteric Defensin 5. Sci Rep. 2016;6:22875. doi: 10.1038/srep22875 26960718 PMC4785407

[9] Scott MG, Vreugdenhil AC, Buurman WA, Hancock RE, Gold MR. Cutting edge: cationic antimicrobial peptides block the binding of lipopolysaccharide (LPS) to LPS binding protein. J Immunol. 2000;164(2):549–53. doi: 10.4049/jimmunol.164.2.549 10623792

[10] Snijder J, van de Waterbeemd M, Glover MS, Shi L, Clemmer DE, Heck AJR. Conformational landscape and pathway of disulfide bond reduction of human alpha defensin. Protein Sci. 2015;24(8):1264–71. doi: 10.1002/pro.2694 25970658 PMC4534177

[11] Szyk A, Wu Z, Tucker K, Yang D, Lu W, Lubkowski J. Crystal structures of human alpha-defensins HNP4, HD5, and HD6. Protein Sci. 2006;15(12):2749–60. doi: 10.1110/ps.062336606 17088326 PMC2242434

[12] Rajabi M, Ericksen B, Wu X, de Leeuw E, Zhao L, Pazgier M, et al. Functional determinants of human enteric α-defensin HD5: crucial role for hydrophobicity at dimer interface. J Biol Chem. 2012;287(26):21615–27. 22573326 10.1074/jbc.M112.367995 PMC3381126

[13] Chapnik N, Levit A, Niv MY, Froy O. Expression and Structure/Function Relationships of Human Defensin 5. Appl Biochem Biotech. 2012;166(7):1703–10. doi: 10.1007/s12010-012-9571-5 22354633

[14] Awang T, Pongprayoon P. The adsorption of human defensin 5 on bacterial membranes: simulation studies. Journal of molecular modeling. 2018;24(10):273. doi: 10.1007/s00894-018-3812-7 30187138

[15] Awang T, Pongprayoon P. The penetration of human defensin 5 (HD5) through bacterial outer membrane: simulation studies. J Mol Model. 2021;27(10):291. doi: 10.1007/s00894-021-04915-w 34546425

[16] Jung SW, Lee J, Cho AE. Elucidating the Bacterial Membrane Disruption Mechanism of Human α-Defensin 5: A Theoretical Study. J Phys Chem B. 2017;121(4):741–8. 28067516 10.1021/acs.jpcb.6b11806

[17] Hsu PC, Jefferies D, Khalid S. Molecular Dynamics Simulations Predict the Pathways via Which Pristine Fullerenes Penetrate Bacterial Membranes. J Phys Chem B. 2016;120(43):11170–9. doi: 10.1021/acs.jpcb.6b06615 27712070

[18] Piggot TJ, Holdbrook DA, Khalid S. Conformational dynamics and membrane interactions of the E. coli outer membrane protein FecA: A molecular dynamics simulation study. Biochimica et Biophysica Acta (BBA)—Biomembranes. 2013;1828(2):284–93. doi: 10.1016/j.bbamem.2012.08.021 22960041

[19] Naughton FB, Kalli AC, Sansom MSP. Association of Peripheral Membrane Proteins with Membranes: Free Energy of Binding of GRP1 PH Domain with Phosphatidylinositol Phosphate-Containing Model Bilayers. The Journal of Physical Chemistry Letters. 2016;7(7):1219–24. doi: 10.1021/acs.jpclett.6b00153 26977543 PMC5593124

[20] Van Der Spoel D, Lindahl E, Hess B, Groenhof G, Mark AE, Berendsen HJ. GROMACS: fast, flexible, and free. Journal of computational chemistry. 2005;26(16):1701–18. doi: 10.1002/jcc.20291 16211538

[21] Zhu F, Hummer G. Convergence and error estimation in free energy calculations using the weighted histogram analysis method. J Comput Chem. 2012;33(4):453–65. doi: 10.1002/jcc.21989 22109354 PMC3271861

[22] Essmann U, Perera L, Berkowitz ML, Darden T, Lee H, Pedersen LG. A smooth particle mesh Ewald method. The Journal of Chemical Physics. 1995;103(19):8577–93.

[23] Hess B. P-LINCS: A Parallel Linear Constraint Solver for Molecular Simulation. Journal of Chemical Theory and Computation. 2008;4(1):116–22. 26619985 10.1021/ct700200b

[24] Humphrey W, Dalke A, Schulten K. VMD: Visual molecular dynamics. Journal of Molecular Graphics. 1996;14(1):33–8. doi: 10.1016/0263-7855(96)00018-5 8744570

[25] Awang T, Chairatana P, Vijayan R, Pongprayoon P. Evaluation of the Binding Mechanism of Human Defensin 5 in a Bacterial Membrane: A Simulation Study. International journal of molecular sciences. 2021;22(22):12401. doi: 10.3390/ijms222212401 34830284 PMC8619297

[26] Simcock PW, Bublitz M, Cipcigan F, Ryadnov MG, Crain J, Stansfeld PJ, et al. Membrane binding of antimicrobial peptides is modulated by lipid charge modification. Journal of Chemical Theory and Computation. 2021;17(2):1218–28. doi: 10.1021/acs.jctc.0c01025 33395285

[27] Zhao J, Zhao C, Liang G, Zhang M, Zheng J. Engineering antimicrobial peptides with improved antimicrobial and hemolytic activities. J Chem Inf Model. 2013;53(12):3280–96. doi: 10.1021/ci400477e 24279498

[28] Necula G, Bacalum M, Radu M. Interaction of Tryptophan-and Arginine-Rich Antimicrobial Peptide with E. coli Outer Membrane—A Molecular Simulation Approach. International Journal of Molecular Sciences. 2023;24(3):2005. doi: 10.3390/ijms24032005 36768325 PMC9916935

[29] Kang X, Elson C, Penfield J, Kirui A, Chen A, Zhang L, et al. Integrated solid-state NMR and molecular dynamics modeling determines membrane insertion of human beta-defensin analog. Commun Biol. 2019;2:402. 31701030 10.1038/s42003-019-0653-6 PMC6825183

[30] Lyu Y, Xiang N, Zhu X, Narsimhan G. Potential of mean force for insertion of antimicrobial peptide melittin into a pore in mixed DOPC/DOPG lipid bilayer by molecular dynamics simulation. The Journal of chemical physics. 2017;146(15):155101. doi: 10.1063/1.4979613 28433027

[31] Catte A, Wilson MR, Walker M, Oganesyan VS. Antimicrobial action of the cationic peptide, chrysophsin-3: a coarse-grained molecular dynamics study. Soft Matter. 2018;14(15):2796–807. doi: 10.1039/c7sm02152f 29595197

[32] Yeasmin R, Brewer A, Fine LR, Zhang L. Molecular Dynamics Simulations of Human Beta-Defensin Type 3 Crossing Different Lipid Bilayers. ACS omega. 2021. doi: 10.1021/acsomega.1c01803 34095684 PMC8173616

[33] Wang C, Zhao G, Wang S, Chen Y, Gong Y, Chen S, et al. A simplified derivative of human defensin 5 with potent and efficient activity against multidrug-resistant Acinetobacter baumannii. Antimicrobial agents and chemotherapy. 2018;62(2): doi: 10.1128/AAC.01504-17 29158275 PMC5786806

[34] Zhang Y, Lu W, Hong M. The membrane-bound structure and topology of a human α-defensin indicate a dimer pore mechanism for membrane disruption. Biochemistry-Us. 2010;49(45):9770–82. 10.1021/bi101512j PMC2992833 20961099

[35] Zhang QY, Yan ZB, Meng YM, Hong XY, Shao G, Ma JJ, et al. Antimicrobial peptides: mechanism of action, activity and clinical potential. Mil Med Res. 2021;8(1):48. doi: 10.1186/s40779-021-00343-2 34496967 PMC8425997

[36] Rodriguez-Rojas A, Baeder DY, Johnston P, Regoes RR, Rolff J. Bacteria primed by antimicrobial peptides develop tolerance and persist. Plos Pathog. 2021;17(3):e1009443. doi: 10.1371/journal.ppat.1009443 33788905 PMC8041211

