# Supplementary figures and images for: Molecular dynamics simulations of human α-defensin 5 (HD5) crossing gram-negative bacterial membrane
Source: PLoS One. 2023 Nov 21;18(11):e0294041. doi: 10.1371/journal.pone.0294041 (PMC10662769; doi:10.1371/journal.pone.0294041)

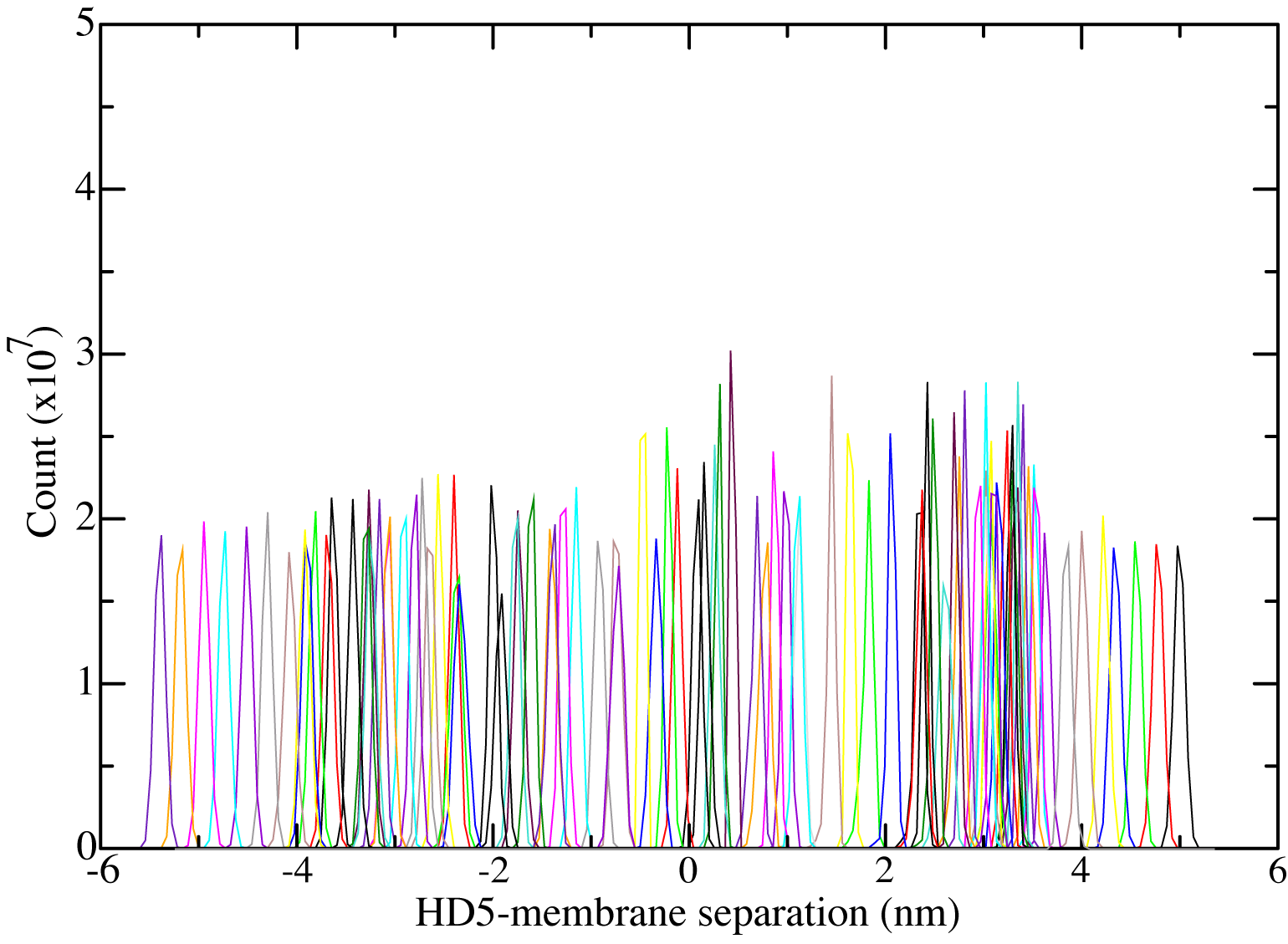

Supplement: S1 Fig — The range is from ~ -5.4 nm to +5.1 nm, which is divided into 92 windows. (TIF) [file pone.0294041.s001.tif]

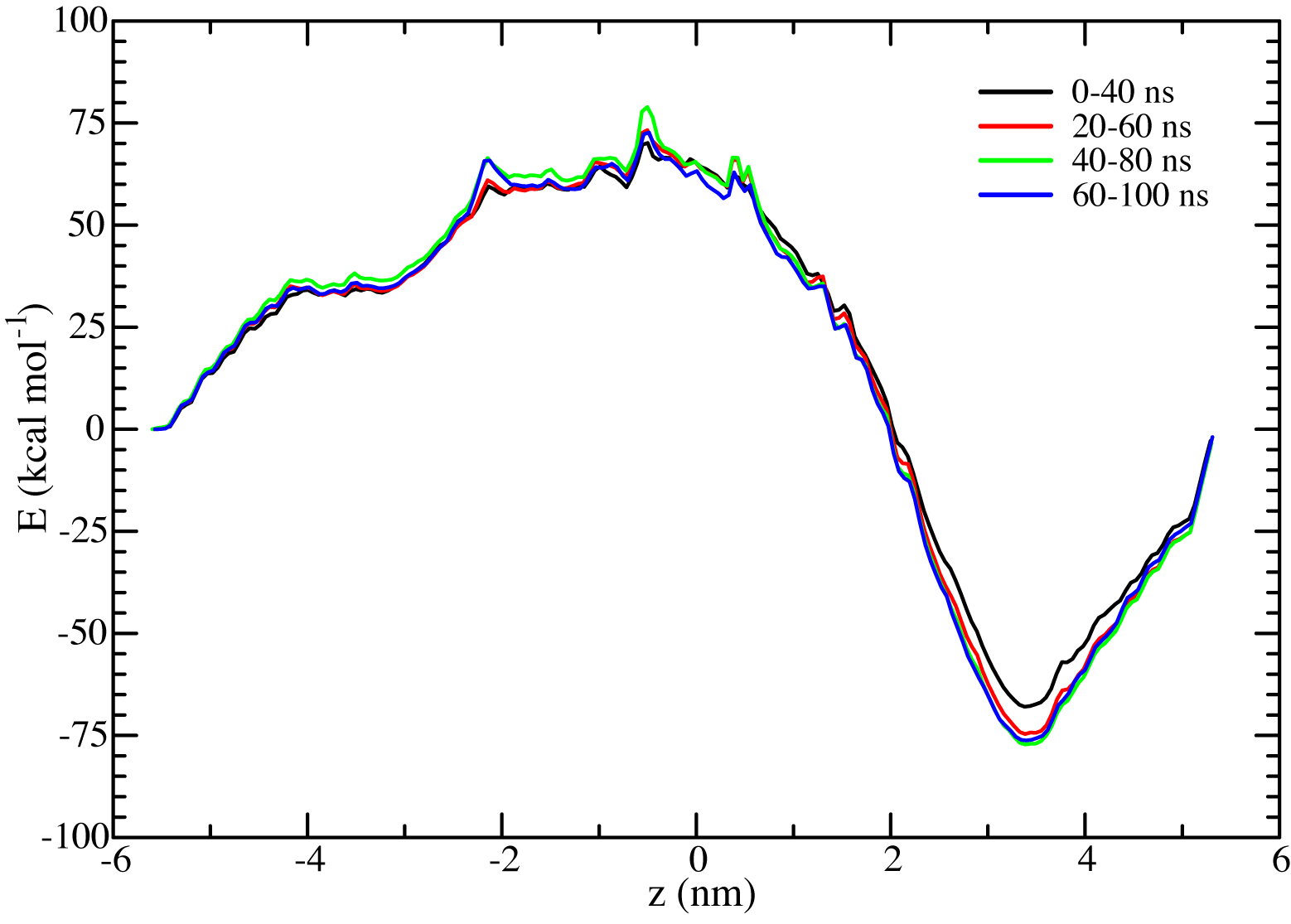

Supplement: S2 Fig — The plots are generated from the data in ranges of 0–40 ns, 20–60 ns, 40–80 ns, and 60–100 ns, respectively. (TIF) [file pone.0294041.s002.tif]

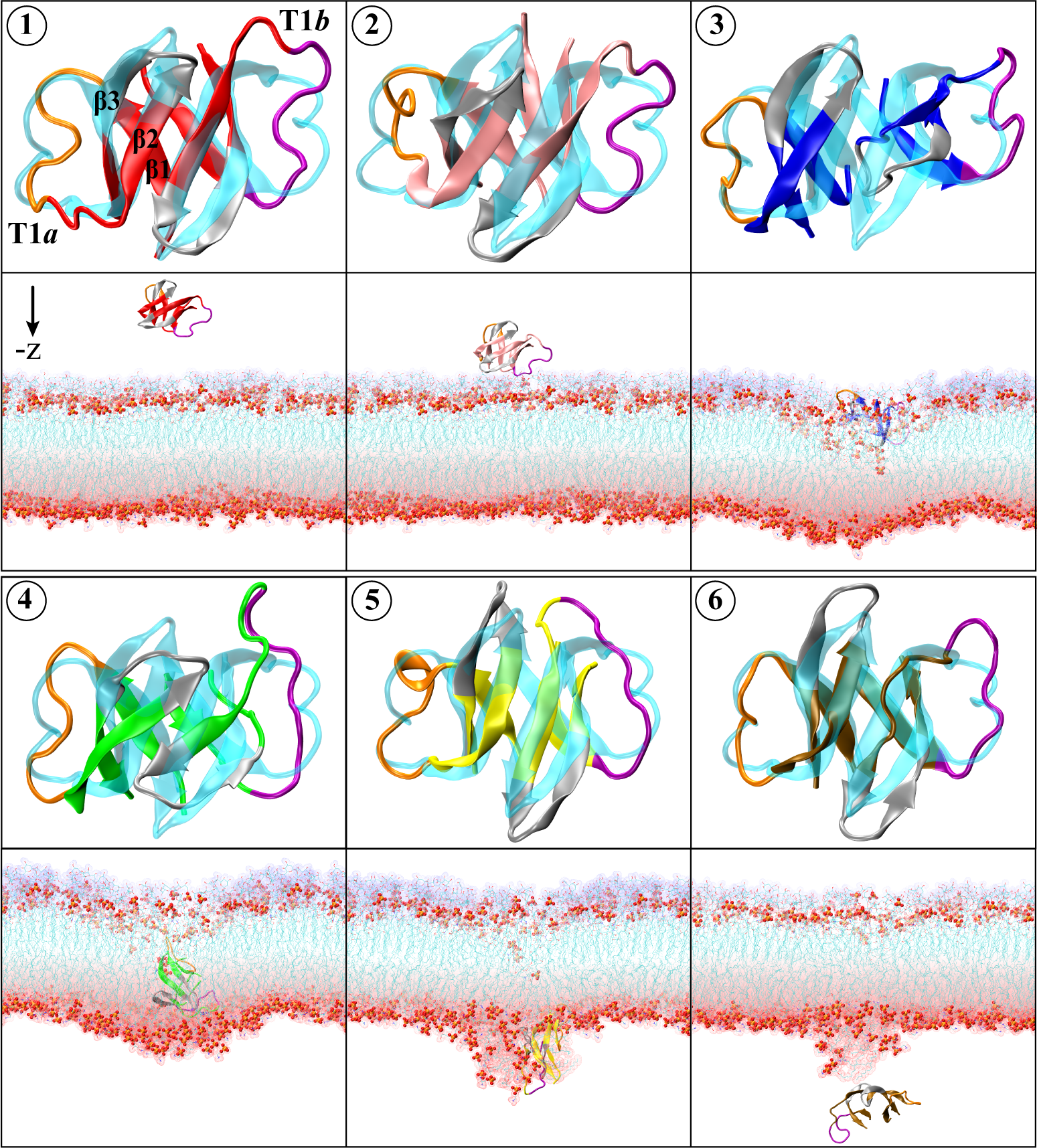

Supplement: S3 Fig — Top: Superimposition of six final snapshots with the X-ray structure of HD5 (PDB ID: 1ZMP, transparent cyan). Bottom: Six stages of HD5 translocation through the LPS membrane. The peptide is shown in cartoon representation, and the lipid head groups are shown in a VDW representation. The lipid tails are shown as lines. The semi-transparent blue and red surfaces correspond to the KDO moieties, Pi(outer) and Pi (inner), respectively. The arrow indicates the direction in which the pulling force is applied: (1) The initial system. (2) The peptide binds to the surface of the outer membrane layer. (3) The peptide buries through the barrier layer of KDO. (4) The peptide reaches the center of the membrane bilayer. (5) The peptide leaves the surface of the inner membrane layer. (6) The peptide is out of the membrane. (TIF) [file pone.0294041.s003.tif]

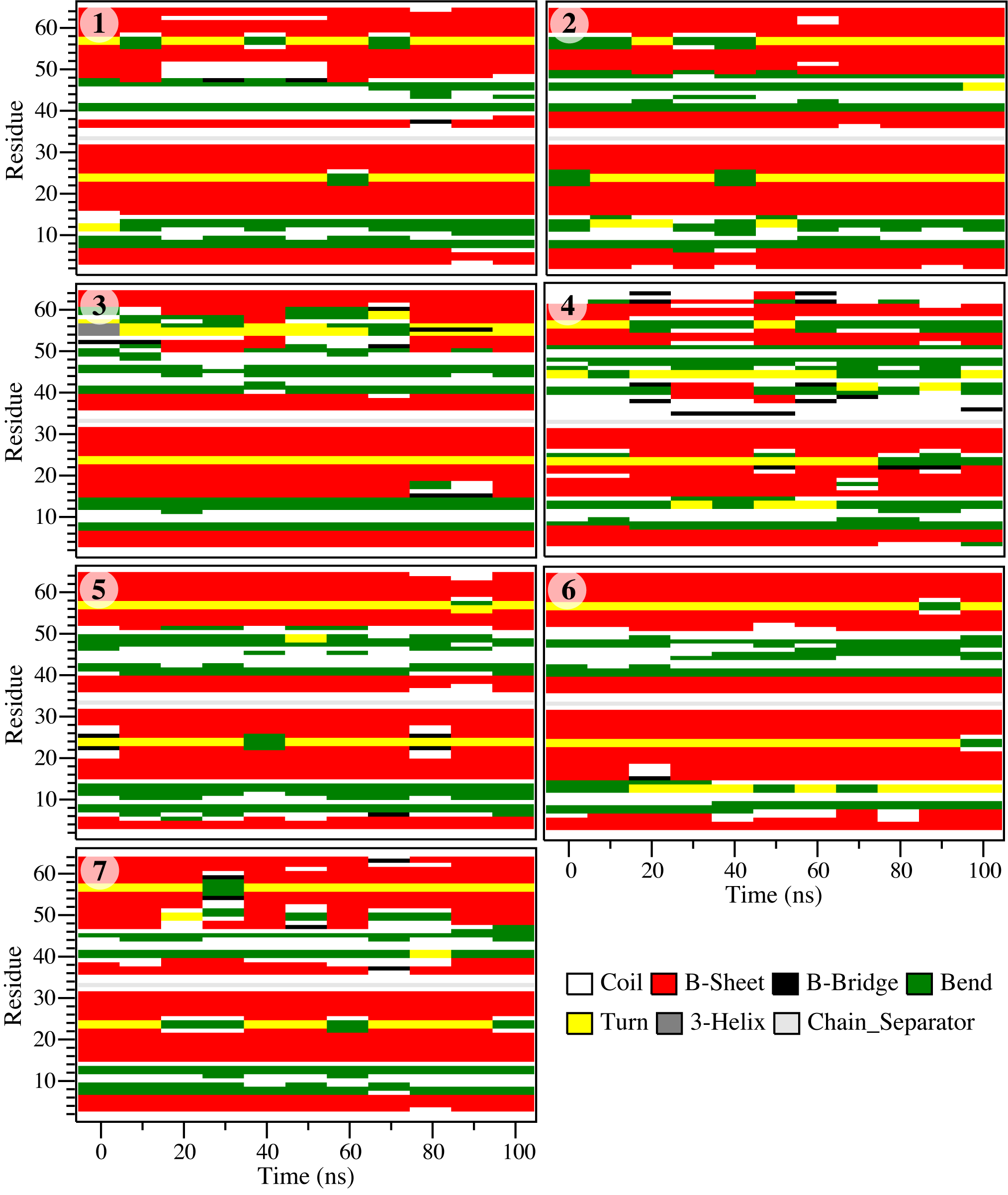

Supplement: S4 Fig — Positions 1–7 are the same as shown in Fig 3B. (TIF) [file pone.0294041.s004.tif]

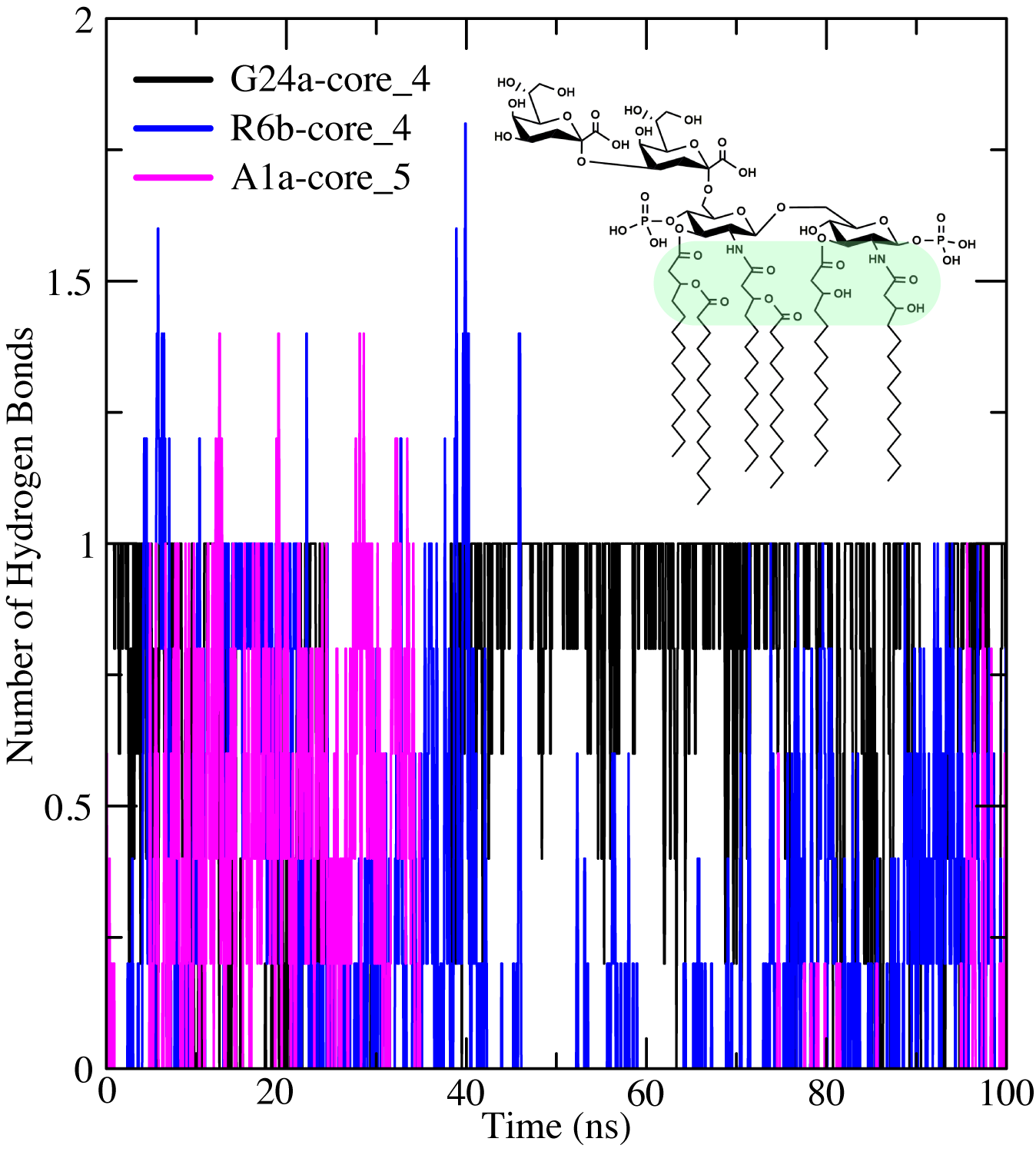

Supplement: S5 Fig — A chemical structure of lipid A is shown as an inset where fatty acid moieties are shown in green band. (TIF) [file pone.0294041.s005.tif]
